# Assessment of Resident Physicians’ Knowledge, Attitudes, and Practices Concerning Advance Care Planning: A Cross-Sectional Study

**DOI:** 10.7759/cureus.86416

**Published:** 2025-06-20

**Authors:** Gloria Y Ojerinde, Kahee A Mohammed

**Affiliations:** 1 Internal Medicine, SSM Health St. Mary's Hospital, St. Louis, USA; 2 Internal Medicine, Saint Louis University - School of Medicine, St. Louis, USA

**Keywords:** advance care planning, advance directive, cultural background, end of life discussions, legal and laws, medical education, religion and culture, resident doctors

## Abstract

Background

Advance Care Planning (ACP) is an essential component of patient-centered care, particularly in end-of-life contexts. This study aimed to assess the knowledge, attitudes, and practices related to ACP among resident physicians at St. Mary’s Hospital, St. Louis, USA, with a focus on perceived skillfulness, comfort level, cultural and religious influences, and perceived barriers to ACP discussions.

Methods

A cross-sectional quantitative study was conducted using an online questionnaire distributed to all Internal Medicine and Family Medicine residents at St. Mary’s Hospital. Of the 48 invited residents, 36 completed the survey. Data were analyzed using descriptive statistics via Excel (Microsoft® Corp., Redmond, WA, USA). Ethical exemption was granted by the Institutional Review Board.

Results

While 61.1% (n = 22) of respondents reported prior training and knowledge of ACP, a significant proportion lacked awareness of state laws and documentation procedures for advance directives. Despite encountering more than five patients weekly, 94.4% (n = 34) had ACP discussions with only one to two patients. Although 86.1% (n = 31) expressed a positive attitude and willingness to engage in ACP discussions, only 33.3% (n = 12) routinely involved family members. Cultural background influenced ACP communication for 41.7% (n = 15) of residents, while 72.2% (n = 26) reported no influence from their religious background. Most residents felt skillful and comfortable discussing core ACP topics, particularly cardiopulmonary resuscitation (CPR), Intensive Care Unit (ICU) admission, and disease prognosis. However, assisting patients in completing advance directives remained a notable area of lower perceived competence. Comfort levels were highest in areas where residents also felt most skilled.

Conclusion

The findings highlight a gap between theoretical knowledge and practical application of ACP, particularly in legal aspects and documentation. Despite positive attitudes and self-reported competence in communication, limited engagement with patients and families indicates a need for more targeted training, especially in legal frameworks and documentation procedures. Enhancing these areas could better equip residents to conduct meaningful and comprehensive ACP discussions.

## Introduction

Advance care planning (ACP) is a structured process, either verbal or written, designed to prepare for periods when a patient is incapable of making independent medical decisions. Evidence demonstrates that ACP enhances patient-centered care, alleviates caregiver burden, and reduces healthcare costs [1]. Physicians are expected to lead ACP discussions, particularly at the end of life, but these conversations are frequently skewed toward medical considerations, potentially neglecting other important domains [2].

Multiple factors influence the effectiveness of ACP discussions between patients and healthcare providers, with cultural and religious backgrounds being particularly significant [3]. Effective communication is essential in ACP, yet standards for information disclosure and the conceptualization of information can differ markedly across cultures. Cultural sensitivity is therefore crucial; discordance between the cultural backgrounds of physicians and patients may result in conflicting perspectives, but culturally sensitive communication can yield better patient outcomes [4].

Systematic reviews have identified racial and ethnic disparities in the provision of palliative care, especially at the end of life in intensive care settings [5]. Research further indicates that the ethno-cultural background of healthcare providers themselves can influence their willingness and approach to discussing end-of-life care. For example, among Israeli-born Jews (Sabras), Israeli Arabs, and Russian immigrants, Sabras were more likely to engage in end-of-life discussions with patients compared to their Arab or Russian colleagues. This difference is attributed to cultural beliefs among Arabs and Russians that discussing death may bring bad luck or increase the risk of harm to patients and their families [3].

Despite the recognized benefits of ACP, many healthcare practitioners lack the necessary knowledge and skills to conduct effective ACP discussions, resulting in suboptimal documentation of advance directives [6]. A national survey among healthcare professionals revealed that 56% had little or no knowledge of the legal framework for ACP, 66% lacked access to appropriate ACP materials for patients, and only 46% performed ACP as necessary. Although 47% reported that ACP discussions were conducted well, and found beneficial, 88% expressed willingness to engage in more ACP discussions in the future [7]. The main barriers identified included a lack of experience and the absence of clear policies or procedures.

Training in ACP correlates with increased confidence among providers in discussing end-of-life care. However, studies indicate that increased knowledge alone does not necessarily translate into greater engagement in ACP discussions. Instead, a combination of high knowledge and a positive attitude is associated with significantly improved engagement [8].

Cross-sectional studies in various countries highlight additional nuances. For example, in the Philippines, 62% of resident physicians reported comfort in discussing ACP, but only 54% were comfortable discussing disease prognosis. Most respondents believed that ACP should be initiated by primary care physicians and that palliative care discussions should occur early, not just when life expectancy is limited [9]. In Japan, physicians working in clinics or nursing homes had less knowledge and less supportive attitudes toward ACP compared to those in other specialties or settings, suggesting that workplace and specialty are significant determinants of ACP-related knowledge and practice [6].

There remains a paucity of research specifically examining the knowledge, attitudes, and practices of ACP among primary care providers, particularly in Internal Medicine and Family Medicine. The present study aims to address these gaps by evaluating perceived knowledge deficits, skill levels, comfort with ACP discussions, and the impact of cultural, religious, or other barriers among resident physicians in primary care specialties.

## Materials and methods

This study utilized a cross-sectional quantitative research design to evaluate resident physicians’ knowledge, attitudes, and practices concerning ACP. The primary objective was to assess physicians' familiarity with ACP concepts, their confidence in initiating end-of-life discussions, and their self-identified need for further training in this area.

Data collection was conducted through a structured, self-administered electronic questionnaire (see Appendix 1). The instrument comprised sections on knowledge of ACP principles, awareness of state and national legal frameworks, self-reported confidence in engaging in ACP conversations with patients and families, and perceived barriers to effective ACP implementation. Additionally, questions explored current ACP-related clinical practices and systemic or interpersonal factors impeding its routine integration into care. Several items in the questionnaire were adapted from a validated national survey previously utilized in a study on ACP among patients with chronic kidney disease [7], thereby enhancing the tool’s content validity and reliability.

The study population consisted of resident physicians from the Internal Medicine and Family Medicine departments at St. Mary’s Hospital in St. Louis, USA. These specialties were purposively selected due to their central roles in primary care, continuity of care, and the high degree of patient interaction, particularly in managing chronic and end-of-life conditions.

A total of 48 resident physicians were eligible to participate in the study. All were invited to complete the survey, which was distributed electronically via Microsoft Forms (Microsoft® Corp., Redmond, WA, USA). Participation was entirely voluntary, and electronic informed consent was obtained prior to survey access. Of those invited, 36 residents (75%) completed the questionnaire. The survey was anonymous to ensure confidentiality and encourage honest reporting. Initial invitations were sent via the institution’s official email system, followed by a reminder email on day 3, and a final follow-up message one week later. Notifications were also reinforced through a secure SMS communication platform.

Data collection was conducted over a six-week period between October and November 2024. Survey responses were extracted and organized using a standardized Microsoft Excel spreadsheet. Data analysis was performed using built-in Excel statistical tools, with descriptive statistics applied to summarize demographic data and response distributions. Inferential analyses, where applicable, were conducted to explore associations between variables of interest, such as level of training and confidence in ACP discussions.

Ethical exemption for this study was granted by the Institutional Review Board (IRB) of St. Mary’s Hospital, as the research posed minimal risk and involved anonymous, informed participation without intervention.

## Results

Demographic characteristics of resident physicians in the Internal Medicine and Family Medicine programs at St. Mary’s Hospital are presented in Table 1, showing the distribution of residency program enrollment, gender, religious affiliation, and ethnicity among participants. A majority of respondents (75.0%, n = 36) were enrolled in the Internal Medicine residency program, and more than half (52.8%, n = 19) identified as female. Regarding religious affiliation, 41.7% (n = 15) of participants identified as Christian. Ethnically, 36.1% (n = 13) of participants reported being of Asian descent.

**Table 1:** Demographic Characteristics of Respondents

Gender	Total (n = 36) (%)	Internal Medicine (n = 26) (%)	Family Medicine (n = 10) (%)
Male	15 (41.7)	10 (27.8)	5 (13.9)
Female	19 (52.8)	14 (38.9)	5 (13.9)
Others	2 (5.6)	2 (5.6)	0 (0)
Religion			
Christian	15 (41.7)	10 (27.8)	5 (13.9)
Islam	5 (13.9)	4 (11.1)	1 (2.8)
Judaism	2 (5.6)	2 (5.6)	0 (0)
Buddhism	0 (0)	0 (0)	0 (0)
Hinduism	6 (16.7)	5 (13.9)	1 (2.8)
Others	1 (2.8)	1 (2.8)	0 (0)
No religion	7 (19.4)	4 (11)	3 (8.3)
Ethnicity			
African/African American participants	9 (25)	8 (22.2)	1 (2.8)
Asian participants	13 (36.1)	10 (27.8)	3 (8.3)
Caucasian participants	9 (25)	3 (8.3)	6 (16.7)
Hispanic Latino participants	2 (5.6)	2 (5.6)	0 (0)
Middle Eastern participants	1 (2.8)	1 (2.8)	0 (0)
Native American participants	0 (0)	0 (0)	0 (0)
Others	2 (5.6)	2 (5.6)	0 (0)

**Figure 1:**
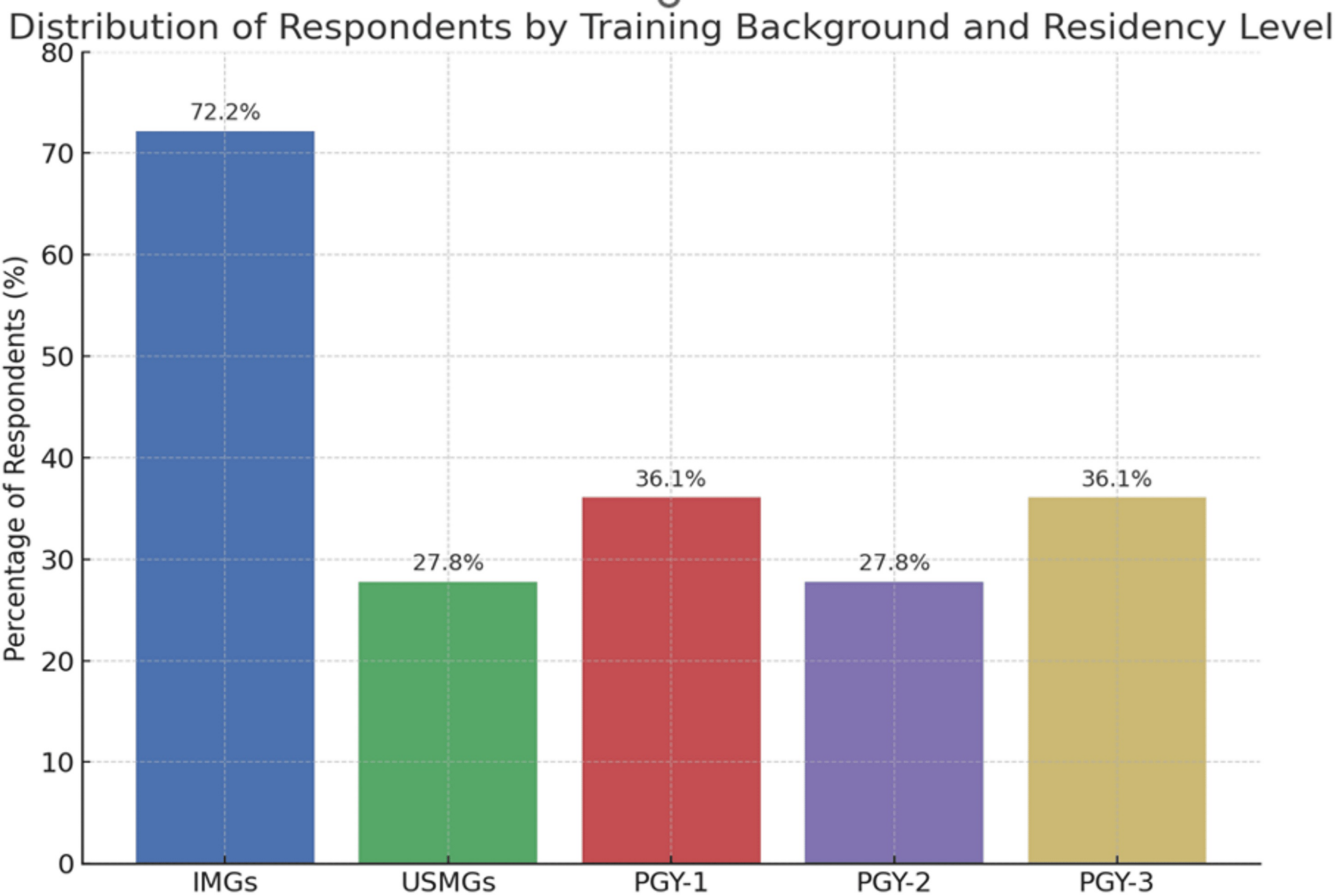
Demography of Respondent IMGs, International Medical Graduates; USMGs, United States Medical Graduates; PGY, Post-graduate Year

Figure 1 is a graphical representation of the respondents. The majority of participants were International Medical Graduates (IMGs), accounting for 72.2% (n = 26). Regarding the level of training, most were Post-graduate Year-1 (PGY-1) and Post-graduate Year-2 (PGY-2) residents, each representing 36.1% (n = 13) of the participants.

Table 2 presents data on clinical workload and ACP practices among respondents. Over half of the participants (58.3%, n = 21) reported attending to more than five patients per day. A majority (61.2%, n = 22) indicated that ACP discussions were conducted on an ad hoc basis, determined by the discretion of individual clinicians. Notably, only 33.3% (n = 12) of respondents reported routinely involving both patients and their families in ACP conversations.

**Table 2:** Current Clinical Practices in ACP ACP, Advance Care Planning

Practices	Total (n = 36) (%)	Internal Medicine (n = 26) (%)	Family Medicine (n = 10) (%)
How many patients do you see on average in the clinic per day?			
A) 3	0 (0)	0 (0)	0 (0)
B) 4-5	15 (41.7)	11 (30.6)	4 (11.1)
C) >5	21 (58.3)	14 (38.9)	7 (19.4)
Out of the above number, how many of these patients did you discuss ACP with?			
A) 1-2	34 (94.4)	25 (69.4)	9 (25)
B) 3-5	2 (5.6)	1 (2.8)	1 (2.8)
C) >5	0 (0)	0	0 (0)
Which of the following most accurately reflects current practice in ACP at your primary workplace?			
A) A formal program of ACP is implemented	0 (0)	0 (0)	0 (0)
B) ACP is carried out on an ad hoc basis at the discretion of an individual clinician	22 (61.2)	16 (44.4)	6 (16.7)
C) ACP never or hardly occurs	7 (19.4)	4 (11.1)	3 (8.3)
D) N/A (I often discuss ACP with my patients)	7 (19.4)	6 (16.7)	1 (2.8)
To what extent do you involve both the patient's family and the patient themselves in conversations about ACP across patient age groups?			
A) All or almost all	8 (22.2)	6 (16.7)	2 (5.6)
B) A Majority	12 (33.3)	10 (27.8)	2 (5.6)
C) A Minority	8 (22.2)	5 (13.9)	3 (8.3)
D) None or Almost None	4 (11.1)	2 (5.6)	2 (5.6)
E) N/A (I do not discuss ACP with my patients in the clinic)	4 (11.1)	3 (8.3)	1 (2.8)

**Figure 2:**
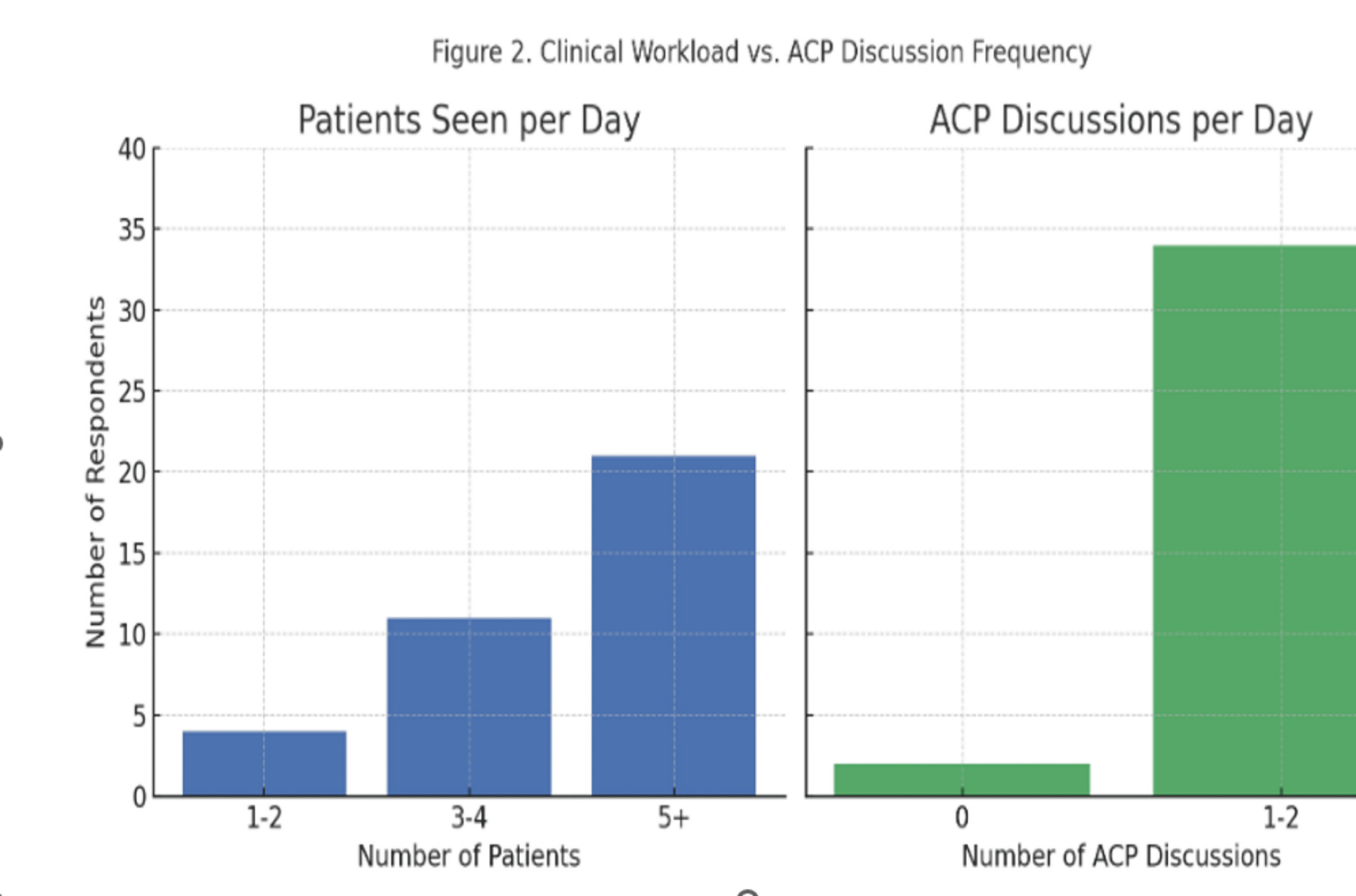
Comparison of Daily Patient Caseload and Frequency of ACP Discussions Among Respondents ACP, Advance Care Planning

As illustrated in Figure 2, the number of patients seen per day in the clinic varied among the 36 respondents. None of the participants (0.0%, n = 0) reported seeing only three patients per day. A total of 15 respondents (41.7%) reported seeing between four and five patients daily, while the majority (21 respondents; 58.3%) indicated seeing more than five patients per day.

With respect to ACP discussions, also depicted in Figure 2, many respondents (34 out of 36; 94.4%) reported engaging in ACP conversations with one to two patients per day. A small proportion (two respondents; 5.6%) reported discussing ACP with three to five patients daily. No respondents (0.0%, n = 0) reported engaging in ACP discussions with more than five patients per day.

Table 3 summarizes respondents’ perceptions and attitudes toward ACP. A large majority (86.1%, n = 31) reported a willingness to engage more frequently in ACP discussions in the future. While 58.3% (n = 21) indicated that their cultural background does not influence their approach to ACP, a notable proportion (41.7%, n = 15) acknowledged that culture plays a significant role in these discussions. Additionally, most respondents (72.2%, n = 26) stated that their religious beliefs do not impact their engagement in ACP conversations with patients.

**Table 3:** Perceptions and Attitudes of Respondents Regarding ACP ACP, Advance Care Planning

Practices	Total (n = 36) (%)	Internal Medicine (n = 26) (%)	Family Medicine (n = 36) (%)
Would you be willing to participate more often in conversations about ACP in the future?			
A) Yes	31 (86.1)	23 (63.9)	8 (22.2)
B) No	0 (0)	0 (0)	0 (0)
C) Not sure	2 (5.6)	1 (2.8)	1 (2.8)
D) N/A (I often discuss ACP with my patients)	3 (8.3)	2 (5.6)	1 (2.8)
Have you ever discussed ACP with people from cultural or linguistic backgrounds other than English?			
A) Yes	23 (63.9)	17 (47.2)	6 (16.7)
B) No	13 (36.1)	9 (25)	4 (11.1)
Does your cultural background influence ACP discussions with your patient?			
A) Yes	15 (41.7)	10 (27.8)	5 (13.9)
B) No	21 (58.3)	16 (44.4)	5 (13.9)
Does your religious belief affect ACP discussions with your patient?			
A) Yes	10 (27.8)	7 (19.4)	3 (8.3)
B) No	26 (72.2)	19 (52.8)	7 (19.4)

Table 4 presents the respondents' self-assessed competencies across various domains of ACP and end-of-life discussions. All participants (100%) reported feeling competent in the communication tasks evaluated, with the exception of "assisting patients in completing an advanced directive," for which a majority expressed a lack of confidence. The domains with the highest perceived competence were those involving discussions about resuscitation preferences, particularly decisions regarding cardiopulmonary resuscitation (CPR), and decisions related to Intensive Care Unit (ICU) admission. These were followed by discussions on disease prognosis, while the lowest levels of perceived competence were observed in conversations addressing death and dying. Competence was measured using a Likert scale ranging from "very unskilled" to "very skilled," with a score range of 1 to 4. For each domain, the mean and standard deviation were calculated to quantify the degree of perceived skillfulness.

**Table 4:** Evaluation of Healthcare Providers’ Perceived Skills in Discussing Advance Care Planning and End-of-Life Issues This table presents the frequency and corresponding percentage (in parentheses) for various topics within the domains of end-of-life care discussions. It also reflects the respondents’ self-reported level of skillfulness in discussing each domain. Additionally, the table includes the mean and standard deviation for each domain, offering a summary of central tendency and variability. CPR, Cardiopulmonary Resuscitation; ICU, Intensive Care Unit

	Very Unskilled (%)	Unskilled (%)	Skilled (%)	Very Skilled (%)	Mean ± SD	Remark
Discussing ACP	1 (2.8)	12 (33.3)	21 (58.3)	2 (5.6)	2.67 ± 0.63	Skilled
Internal Medicine	0 (0)	9 (25)	15 (41.7)	2 (5.6)	2.73 ± 0.59	Skilled
Family Medicine	1 (2.8)	3 (8.3)	6 (16.7)	0 (0)	2.50 ± 0.68	Skilled
Assisting patients to complete an advanced directive	2 (5.6)	23 (63.9)	9 (25.0)	2 (5.6)	2.31 ± 0.67	Unskilled
Internal Medicine	1 (2.8)	18 (50)	6 (16.7)	1 (2.8)	2.22 ± 0.61	Unskilled
Family Medicine	0 (0)	5 (13.9)	3 (8.3	1 (2.8)	2.55 ± 0.54	Unskilled
Discussing disease prognosis	1 (2.8)	5 (13.9)	25 (69.4)	5 (13.9)	2.94 ± 0.63	Skilled
Internal Medicine	1 (2.8)	3 (8.3)	18 (50)	4 (11.1)	2.96 ± 0.64	Skilled
Family Medicine	0 (0)	2 (5.6)	7 (19.4)	1 (2.8)	2.90 ± 0.53	Skilled
Discussing death and dying	0 (0)	14 (38.9)	16 (44.4)	6 (16.7)	2.78 ± 0.72	Skilled
Internal Medicine	0 (0)	12 (33.3)	10 (27.8)	4 (11.1)	2.69 ± 0.71	Unskilled
Family Medicine	0 (0)	2 (5.6)	6 (16.7)	2 (5.6)	3.00 ± 0.77	Skilled
Discussing potential future withdrawal or withholding life-prolonging medical interventions	3 (8.4)	8 (22.2)	22 (61.1)	3 (8.4)	2.61 ± 0.77	Skilled
Internal Medicine	3 (8.4)	6 (16.7)	15 (41.7)	2 (5.6)	2.46 ± 0.80	Skilled
Family Medicine	0 (0)	2 (5.6)	7 (19.4)	1 (2.8)	2.90 ± 0.53	Skilled
Discussing whether to attempt or not to attempt CPR or go to the ICU	0	4 (11.1)	27 (75.0)	5 (13.9)	3.03 ± 0.51	Skilled
Internal Medicine	0 (0)	4 (11.1)	18 (50)	4 (11.1)	3.00 ± 0.54	Skilled
Family Medicine	0 (0)	0 (0)	9 (25)	1 (2.8)	3.10 ± 0.30	Skilled

Table 5 presents respondents’ self-reported comfort levels in discussing various ACP and end-of-life topics with patients, based on responses from a total of 36 participants. A four-point Likert scale was used to assess comfort levels, ranging from 1 (very uncomfortable) to 4 (comfortable). Mean and standard deviation values were calculated to quantify the overall level of comfort across different domains.

**Table 5:** Evaluation of Healthcare Providers’ Level of Comfort in Discussing Advance Care Planning and End-of-Life Issues This table presents the frequency and corresponding percentage (in parentheses) for various end-of-life care discussion topics, along with respondents’ reported level of comfort in discussing each domain. The table also includes the mean and standard deviation for each domain, summarizing central tendency and variability. CPR, Cardiopulmonary Resuscitation; ICU, Intensive Care Unit

	Very Uncomfortable (%)	Uncomfortable (%)	Comfortable (%)	Very Comfortable (%)	Mean ± SD	Remark
Disease prognosis	1 (2.8)	6 (16.7)	22 (61.1)	7 (19.4)	2.97 ± 0.70	Comfortable
Internal Medicine	1 (0)	4 (11.1)	17 (47.2)	4 (11.1)	3.57 ± 0.92	Comfortable
Family Medicine	0 (0)	2 (5.6)	5 (13.9)	3 (8.3)	3.10 ± 0.70	Comfortable
Death or dying	1 (2.8)	15 (41.7)	14 (38.9)	6 (16.7)	2.69 ± 0.79	Uncomfortable
Internal Medicine	1 (2.8)	12 (33.3)	10 (27.8)	3 (8.3)	2.57 ± 0.73	Uncomfortable
Family Medicine	0 (0)	3 (8.3)	4 (11.1)	3 (8.3)	3.00 ± 0.77	Comfortable
Potential future withdrawal or withholding	4 (11.1)	6 (16.7)	18 (50.0)	8 (22.20)	2.42 ± 0.65	Comfortable
Internal Medicine	4 (11.1)	4 (11.1)	13 (36.1)	5 (13.9)	2.73 ± 0.93	Comfortable
Family Medicine	0 (0)	2 (5.6)	5 (13.9)	3 (8.3)	3.10 ± 0.70	Comfortable
Whether or not to attempt CPR or ICU	1 (2.8)	3 (8.3)	22 (61.1)	10 (27.8)	3.14 ± 0.68	Comfortable
Internal Medicine	1 (2.8)	3 (8.3)	15 (41.7)	7 (19.4)	3.07 ± 0.72	Comfortable
Family Medicine	0 (0)	0 (0)	7 (19.4)	3 (8.3)	3.33 ± 0.45	Comfortable

Overall, respondents reported a general sense of comfort in discussing the listed ACP topics. The highest mean comfort level was observed for discussions regarding the initiation of CPR or admission to the ICU (mean = 3.14), followed by discussions concerning disease prognosis (mean = 2.97).

Table 6 presents the self-reported barriers to conducting ACP discussions among resident physicians, based on responses from 36 participants. Barriers were assessed using a three-point Likert scale, where 1 indicated “not a barrier,” 2 indicated a “minor barrier,” and 3 indicated a “substantial barrier.” Mean scores and standard deviations were calculated to evaluate the perceived impact of each barrier.

**Table 6:** Factors Impeding the Implementation of ACP ACP, Advance Care Planning

	Not a Barrier	Minor Barrier	Substantial Barrier	Mean ± SD	Remark
Lack of time	2 (5.6)	8 (22.2)	26 (72.2)	2.67 ± 0.59	Substantial barrier
Internal Medicine	1 (2.8)	7 (19.4)	18 (50)	2.65 ± 0.54	Substantial barrier
Family Medicine	1 (2.8)	1 (2.80)	8 (22.2)	2.70 ± 0.64	Substantial barrier
Patient and family discomfort	8 (22.2)	18 (50)	10 (27.8)	2.08 ± 0.73	Minor barrier
Internal Medicine	7 (19.4)	11 (30.6)	8 (22.2)	1.26 ± 1.08	Minor barrier
Family Medicine	1 (2.8)	7 (19.4)	2 (5.6)	2.40 ± 0.64	Minor barrier
Healthcare professionals' discomfort in discussing end-of-life care	10 (27.8)	21 (58.3)	5 (13.9)	1.86 ± 0.64	Minor barrier
Internal Medicine	7 (19.4)	16 (44.4)	3 (8.3)	1.84 ± 0.59	Minor barrier
Family Medicine	3 (8.3)	5 (13.9)	2 (5.6)	1.90 ± 0.70	Minor barrier
Difficulty involving family members is especially due to family dynamics	5 (13.9)	18 (50.0)	13 (36.1)	2.22 ± 0.68	Minor barrier
Internal Medicine	3 (8.3)	14 (38.9)	9 (25)	2.23 ± 0.63	Minor barrier
Family Medicine	2 (5.6)	4 (11.1)	4 (11.1)	2.20 ± 0.74	Minor barrier
Lack of policy or procedures for ACP	5 (14.3)	14 (45.7)	17 (40)	2.28 ± 0.70	Minor barrier
Internal Medicine	4 (11.1)	11 (30.6)	11 (30.6)	2.26 ± 0.70	Minor barrier
Family Medicine	1 (2.8)	3 (8.3)	6 (16.7)	2.50 ± 0.67	Substantial barrier
Environmental problems (lack of space)	20 (54.3)	14 (40)	2 (5.7)	1.56 ± 0.65	Not a barrier
Internal Medicine	14 (38.9)	10 (27.8)	2 (5.6)	1.53 ± 0.63	Not a barrier
Family Medicine	6 (16.7)	4 (11.1)	0 (0)	1.40 ± 0.48	Not a barrier
Cultural and language barriers	12 (33.3)	14 (38.9)	10 (27.8)	1.94 ± 0.79	Minor barrier
Internal Medicine	10 (27.8)	9 (25)	7 (19.4)	1.88 ± 0.79	Minor barrier
Family Medicine	2 (5.6)	5 (13.9)	3 (8.3)	2.10 ± 0.70	Minor barrier
Lack of access to materials about ACP for my patient	7 (19.4)	16 (44.4)	13 (36.1)	2.17 ± 0.74	Minor barrier
Internal Medicine	5 (13.9)	12 (33.3)	9 (25)	2.15 ± 0.71	Minor barrier
Family Medicine	2 (5.6)	4 (11.1)	4 (11.1)	2.20 ± 0.74	Minor barrier

Among the listed factors, the lack of time was identified as the most significant barrier to ACP discussions, with the highest mean score of 2.67, suggesting it is perceived as a substantial obstacle. In comparison, other factors were considered less impactful, with the absence of formal institutional policies or procedures for ACP rated as the most notable among these, receiving a mean score of 2.28.

**Figure 3:**
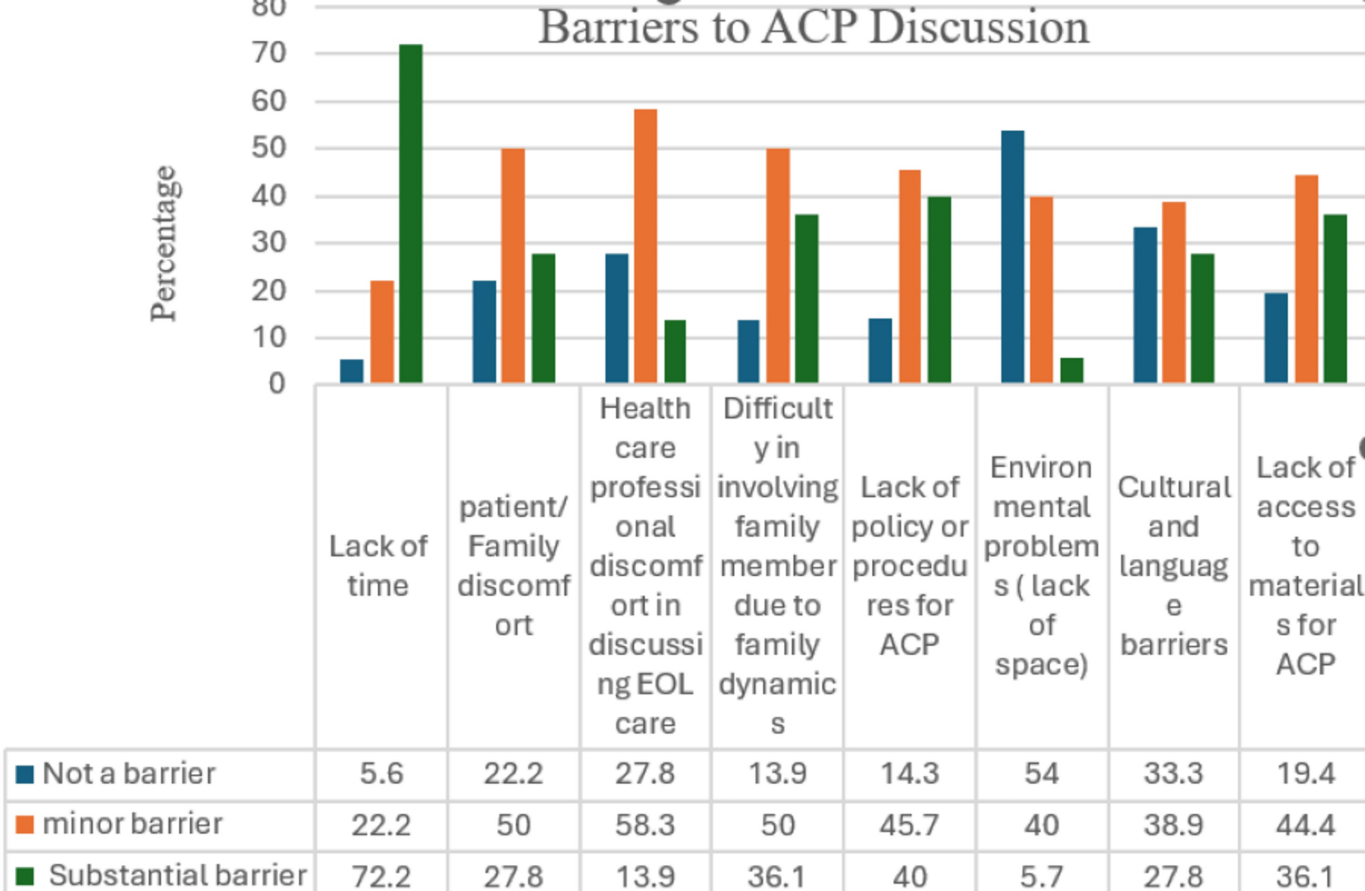
Factors Impeding the Implementation of ACP This figure presents a histogram illustrating the perceived barriers to ACP discussions. The y-axis represents the percentage of respondents, while the x-axis denotes the various identified barriers. ACP, Advance Care Planning; EOL, End-of-Life

Figure 3 presents a histogram depicting the barriers to ACP implementation as reported by respondents. The bars are color-coded to indicate the degree of influence each barrier has on ACP discussions. The blue bar represents "not a barrier," the orange bar indicates a "minor barrier," and the green bar signifies "substantial barriers." The x-axis represents the identified barriers, while the y-axis illustrates the percentage of respondents indicating involvement with each barrier.

## Discussion

This study provides important insights into the knowledge, attitudes, and practices surrounding ACP among Internal Medicine and Family Medicine resident physicians. Although most respondents (61.1%, n = 22) reported prior exposure to ACP-related education or training, a persistent and substantial gap remains between knowledge acquisition and the consistent integration of ACP into routine clinical practice. These findings underscore enduring challenges in medical education, the practical implementation of ACP, and compliance with relevant legal and institutional frameworks.

A particularly salient finding was the limited awareness of legal requirements governing ACP documentation, with 83% of residents unfamiliar with applicable laws. Accurate and legally compliant documentation of advance directives is not only a clinical obligation but also a legal necessity, central to safeguarding patient autonomy in future healthcare decisions. This deficit in legal literacy mirrors the findings of Frandsen et al., who reported that 56% of healthcare professionals lacked sufficient legal and procedural knowledge to conduct effective ACP discussions [7].

Moreover, the study revealed a marked disconnect between ACP-related knowledge and its application in clinical contexts. Despite prior training, few residents routinely initiated ACP conversations. Specifically, 94.4% (n = 34) reported discussing ACP with only one or two patients per clinic session, despite typically seeing five or more patients. Furthermore, 19.4% (n = 7) acknowledged that such discussions were infrequent. These findings align with those of Payongayong et al., who demonstrated that knowledge alone is insufficient to drive behavioral change [8]. Barriers to implementation likely include limited time, competing clinical responsibilities, lack of confidence, minimal experience, and the absence of institutional support or standardized protocols.

Notably, residents reported that they had greater comfort addressing discrete ACP components, such as code status and prognosis, yet expressed limited confidence in facilitating the formal completion of advanced directives. This discrepancy likely reflects procedural uncertainty and logistical barriers, such as constrained appointment times and insufficient administrative infrastructure. These results diverge slightly from those reported by Sachiko et al., where 62% of participants were generally comfortable discussing ACP, but only 54% felt confident addressing prognosis, highlighting the nuanced variability in clinician comfort across ACP domains [9].

Time constraints emerged as the most frequently cited barrier to effective ACP implementation. The complexity of clinical care, high patient volumes, and administrative demands frequently preclude the opportunity for meaningful, in-depth ACP conversations. The lack of structured workflows and dedicated ACP protocols further impedes integration. These challenges underscore the need for scalable and sustainable institutional strategies, such as scheduling dedicated ACP visits, utilizing electronic documentation tools, and incorporating trained facilitators or care coordinators to support ACP discussions without increasing primary care providers’ workloads.

Cultural and linguistic factors also played a significant role in shaping ACP engagement. While 58.3% (n = 21) of residents reported that their own cultural background did not influence their ACP approach, 41.7% (n = 15) acknowledged a potential impact. These findings are consistent with those of Elaine [4], who emphasized the importance of cultural sensitivity in end-of-life communication. Given the high proportion of IMGs in the cohort, cross-cultural dynamics likely influence physician attitudes and communication styles. Cultural incongruence between physicians and patients may contribute to discomfort, miscommunication, or avoidance of sensitive conversations, as supported by Balin et al., who found that clinicians’ cultural backgrounds significantly affect their willingness and approach to ACP discussions [3].

Language barriers were another notable obstacle, with 22.2% (n = 8) of residents citing difficulty engaging non-English-speaking patients in ACP conversations. This finding is in line with prior work by Puerto et al., which demonstrated that limited English proficiency is a significant barrier to ACP engagement and contributes to disparities in care. These results highlight the need for institutional resources, such as professional medical interpreters, culturally tailored educational materials, and multilingual ACP tools, to ensure equitable access across diverse patient populations [10].

Interestingly, the personal religious beliefs of resident physicians were not widely perceived as impediments to ACP discussions. The majority (72.2%, n = 26) reported no conflict between their religious views and the facilitation of ACP, reflecting a commendable degree of professional neutrality and commitment to patient-centered care. Nevertheless, clinicians must remain attentive to patients’ religious beliefs, which often significantly influence preferences around life-sustaining treatments and end-of-life decisions. These findings are consistent with Bowman et al., who found that healthcare providers’ religious affiliations were not major barriers to ACP engagement [11].

Limitations and future directions

Several limitations should be acknowledged when interpreting the findings of this study. First, the sample size was relatively small and drawn from a single community-based residency program, which limits generalizability. Additionally, the focus on resident physicians may not reflect the perspectives or practices of more experienced clinicians, such as fellows or attending physicians.

Future research should aim to include larger, more diverse cohorts, spanning multiple institutions and geographic regions, to enhance external validity. Expanding the sample to include physicians at different stages of training and practice could provide a more comprehensive understanding of ACP implementation across the continuum of care.

Another important limitation is the predominance of IMGs in the study cohort, many of whom completed their undergraduate medical education outside the United States. Comparative studies investigating differences in ACP-related knowledge, attitudes, and behaviors between IMGs and U.S. medical graduates may yield valuable insights into the influence of educational and cultural background on ACP engagement. Such findings could inform the development of targeted curricula and institutional policies designed to close identified gaps and promote more consistent, legally sound, and culturally sensitive ACP practices.

## Conclusions

This study highlights significant gaps between resident physicians’ knowledge, attitudes, and actual practices regarding ACP within Internal Medicine and Family Medicine training programs. While most residents report prior training and express positive attitudes toward ACP, these do not consistently translate into routine clinical practice. Critical barriers, such as insufficient knowledge of legal frameworks (especially in the area of advance directive documentation requirements), time constraints, lack of institutional protocols, and cultural or linguistic discordance with patients, impede the effective delivery of ACP.

The results suggest that, while educational interventions have improved theoretical understanding and general willingness, these are insufficient alone to drive consistent practice change. Instead, a multifaceted approach is required to address this - one that includes targeted training on legal documentation, system-level integration of ACP workflows, and culturally competent care delivery. These findings further affirm the need for residency programs to adopt structured ACP curricula that emphasize both cognitive and practical competencies, in alignment with national goals for patient-centered, values-based care.

## Appendices

Appendix 1: Questionnaire

Knowledge, Attitudes, and Practices of Advance Care Plan Among Resident Doctors

Demography

PGY-Level

A) PGY-1

B) PGY-2

C) PGY-3

Specialty

A) Internal medicine 

B) Family medicine 

Gender

A) Male 

B) Female 

C) Others

Religion

A) Christianity

B) Islam

C) Judaism 

D) Buddhism

E) Hinduism 

F) Others 

G) No religion

Ethnicity

A) African/ African American

B) Asian

C) Caucasian 

D) Hispanic Latino

E) Middle Eastern

F) Native American

G) Others

Place of Medical School

A) United States

B) International Medical graduates (IMG)

Knowledge Assessment Amongst Respondents

Have you received any training in ACP discussions?

A) Yes

B) No

If yes to the above, what was the form of teaching /training?

A) Online

B) Attended lecture and workshop

C) Small group experiential 

D) Simulated patient

E) Role plays in small groups

F) Mentoring from colleagues

G) Feedback from supervisors/ Mentor

H) Others

Are you aware of the state and national legal framework for ACP?

A) Yes

B) No

Are you aware of any available templates in your clinic for ACP discussion?

A) Yes

B) No

Which of these statements about ACP is accurate?

A) It’s a one-time discussion followed by documentation of patient plan

B) It is a continuous process that involves a series of interactions and is amiable to change

Is it inappropriate to discuss advanced directives when a patient is stable?

A) Yes

B) No

If not, state your reason.

Long answer text _________________

Assessment of Current Practices of ACP

Description (optional)

How many patients do you see on average in the clinic per day?

A) 3

B) 4-5

C) >5

Out of the above number, how many of these patients did you discuss ACP with?

A) 1-2

B) 3-5

C) >5

Which of the following most accurately reflects current practice in ACP at your primary workplace?

A) A formal program on ACP is implemented

B) ACP is carried out on an ad hoc basis at the discretion of individual clinician

C) ACP never or hardly occurs

D) Unsure

To what extent do you involve both the patient's family and patient themselves in conversations about ACP across patient age groups?

A) All or almost all

B) A Majority

C) A Minority

D) None or Almost None

E) N/A (I do not discuss ACP with my patients in the clinic)

What factor(s) do you believe will influence readdressing previously documented ACP? (List at least two)

Long answer text_____________________

Would you be willing to participate more often in conversations about ACP in the future?

A) Yes

B) No

C) Not sure

D) N/A (I often discuss ACP with my patients)

Have you ever discussed ACP with people from cultural or linguistic backgrounds other than English?

A) Yes

B) No

If not, why?

Long answer text_____________________

Does your cultural background influence ACP discussion with your patient?

A) Yes

B) No

Does your religious belief affect ACP discussion with your patient?

A) Yes

B) No

Please indicate how skilled (Very Unskilled, Unskilled, Skilled, very Skilled) you feel, or would feel in doing the following with your patients

   Description (optional)

   Discussing ACP

   Assisting patients to complete an advanced directive

   Discussing disease prognosis

   Discussing death and dying

   Discussing potential future withdrawal or withholding life prolonging medical interventions

   Discussing whether to attempt or not attempt CPR or go to the ICU

Please indicate how comfortable (Very Uncomfortable, Uncomfortable, Comfortable, very comfortable) you feel, or would feel discussing the following with your patient

   Description (optional)

   Discussing ACP

   Assisting patients to complete an advanced directive

   Discussing disease prognosis

   Discussing death and dying

   Discussing potential future withdrawal or withholding of life-prolonging medical interventions

   Discussing whether to attempt or not to attempt CPR or go to the ICU

   Barriers to ACP implementation.

Please rate the degree (Not a Barrier, A Minor Barrier, A Substantial Barrier) to which you perceive the following to be a barrier to ACP at your workplace.

   Lack of time

   Patient /Family discomfort

   Health care professional discomfort in discussing EOL care

   Difficulty involving family members especially due to family dynamics

   Lack of policy or procedures for ACP

   Environmental problems (Lack of space)

   Cultural and language barriers

   Lack of access to materials about ACP for my patients
